# Low-frequency perfect sound absorption achieved by a modulus-near-zero metamaterial

**DOI:** 10.1038/s41598-019-49982-5

**Published:** 2019-09-17

**Authors:** Chen Shao, Houyou Long, Ying Cheng, Xiaojun Liu

**Affiliations:** 1 Key Laboratory of Modern Acoustics, Department of Physics and Collaborative Innovation Center of Advanced Microstructures, Nanjing University, Nanjing, 210093 China; 2 State Key Laboratory of Acoustics, Institute of Acoustics, Chinese Academy of Sciences, Beijing, 100190 China

**Keywords:** Acoustics, Metamaterials

## Abstract

We have analytically proposed a mechanism for achieving a perfect absorber by a modulus-near-zero (MNZ) metamaterial with a properly decorated imaginary part, in which the perfect absorption (PA) is derived from the proved destructive interference. Based on the analysis, an ultrathin acoustic metamaterial supporting monopolar resonance at 157 Hz (with a wavelength about 28 times of the metamaterial thickness) has been devised to construct an absorber for low-frequency sound. The imaginary part of its effective modulus can be easily tuned by attentively controlling the dissipative loss to achieve PA. Moreover, we have also conducted the experimental measurement in impedance tube, and the result is of great consistency with that of analytical and simulated ones. Our work provides a feasible approach to realize PA (>99%) at low frequency with a deep-wavelength dimension which may promote acoustic metamaterials to practical engineering applications in noise control.

## Introduction

In recent years, acoustic artificial metamaterials with exotic effective parameters have great advances in manipulating sound waves, such as acoustic cloaking^1^, acoustic emssion^2^, acoustic negative refraction^3,4^, subwavelength imaging^5,6^, wave-front modulation^7–9^, and topological acoustics^10–12^. Traditional absorbers such as porous materials necessitate a thick absorbing material when working at low-frequency range^13^. Limited by such critical issues, extensive studies have been focused on sound absorption at low-frequency through the development of acoustic metamaterials. Li *et al*.^14^ presented a metasurface-based perfect absorber capable of achieving the total absorption of the acoustic waves in an extremely low frequency region. Perfect acoustic absorbers via spiral metasurfaces composed of coiled channels and embedded apertures were present with an ultra-thin thickness down to ∼1/100th of the operating wavelength^15^. Yang *et al*. proposed a general recipe for causal optimality in sound absorption with Fabry–Pérot channels and achieved broadband near-perfect absorption spectrum starting at 400 Hz^16^. Based on localized resonances, different types of resonant elements can exhibit distinct abnormal responses which are denoted as physical negative constituent parameters, i.e., some elements with dipolar resonance^17^ such as acoustic membranes^18–20^ and rubber-coated solid spheres^21^ can generate negative effective mass density, while other elements with monopolar resonance^17^ such as Helmholtz^22–29^ resonators show negative effective bulk modulus. For example, membrane metamaterials with a negative effective mass density can almost totally absorb low-frequency sound waves with a deep subwavelength^30,31^. In addition, Mie resonator^17^ can generate negative effective bulk modulus and negative effective density at multi-frequency bands since it supports multi-order monopoles and dipoles. The effective parameters of the metamaterial are highly dependant on the geometry of the structure based on the basic resonant elements. The conception of double-negative^32,33^ or double-zero^34^ effective parameters has been developed and adapted in the absorption of sound waves. Duan *et al*.^35^ have theoretically realized the perfect absorption (PA) of elastic waves with single-zero-index metamaterials. However, little work has been devoted to utilizing the modulus-near-zero acoustic metamaterial absorbers to fulfill PA at a deep sub-wavelength scale.

In this work, inspired by the studies on permeability-near-zero metamaterials^36,37^ in electromagnetic counterpart, modulus-near-zero metamaterials have been utilized to achieve PA in acoustics. We analytically deduce, and numerically demonstrate that the metamaterial absorber (MA) with a vanishing real part and an appropriate imaginary part of bulk modulus can acquire PA. Moreover, we have designed and fabricated a metamaterial with a purely imaginary bulk modulus at subwavelength dimensions in a single configuration, which makes it capable of many applications in eliminating low-frequency noise. By controlling the channel width of the metamaterial structure, the dissipation loss (related to the imaginary part of the bulk modulus) can be adapted to fulfill the PA condition. In contrast to the previous work by Long *et al*.^38^, which focused on multiband quasi-perfect absorption by satisfying near critical coupling condition, here we develop a general theory to get PA with the MNZ MAs at designed working frequency. In addition, we would like to note that by using two critically coupled Mie resonators to achieve monopolar and dipolar resonances respectively in the same frequency window, double-zero parameter for the design of the perfect absorber can be envisioned, which may provide a ventilated absorption system.

## Results

### Theoretical results

**Figure 1:**
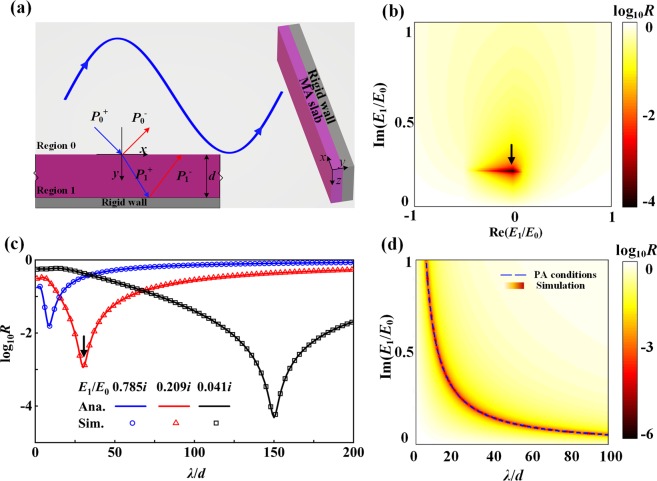
(**a**) Configuration of the theoretical model. The metamaterial absorber (MA) slab in deep sub-wavelength thickness $d\ll \lambda$ is located between the background medium and rigid wall. The reflected waves exhibit π phase jump at the front surface and zero reflection phase at the rear surface, respectively. (**b**) Map of theoretical reflectance *R* in dependence on the real and imaginary parts of *E*_1/_*E*_0_ at slab thickness *d* = *λ*/30, where *E*_1_ and *E*_0_ are the effective bulk modulus of MA slab and background medium, respectively. (**c**) Reflectance *R* for *E*_eff_/*E*_0_ = 0.041*i*, 0.209*i* and 0.785*i* at different *λ*/*d* with *λ* fixed. (**d**) Reflectance *R* in dependence on the imaginary part of *E*_1_/*E*_0_ and *λ*/*d* with *λ* fixed. The simulations confirm the analytical predictions.

In order to clarify the design idea, the analytical formulas are developed to clarify the mechanism and derive theoretical requirements to achieve PA for the modulus-near-zero (MNZ) MA. Figure 1(a) plots the configuration of the theoretical model, which is composed of air, the ultra-thin slab, and a rigid backing wall. The background medium air and the MA slab with a realistic geometric thickness *d* are denoted as regions 0 and 1, respectively. *ρ*_0_ and *ρ*_1_ are the densities of the air and the slab while *E*_0_ and *E*_1_ are the corresponding bulk modulus. From the wave equation, the pressure field and velocity field can be expressed as

1 $$\{\begin{array}{c}{V}_{ny}=({k}_{ny}/\omega {\rho }_{n})({p}_{n}^{+}{e}^{-i{k}_{ny}y}-{p}_{n}^{-}{e}^{i{k}_{ny}y}){e}^{-i{k}_{nx}x}\\ {V}_{nx}=({k}_{nx}/\omega {\rho }_{n})({p}_{n}^{+}{e}^{-i{k}_{ny}y}+{p}_{n}^{-}{e}^{i{k}_{ny}y}){e}^{-i{k}_{nx}x}\end{array},$$

where ${p}_{n}^{+}$ and ${p}_{n}^{-}$ are the pressure field amplitudes of the incident plane waves and the reflective waves in the region *n* (*n* = 0 and 1), respectively. By applying boundary conditions at the interfaces (the continuities of the pressure field and the vertical components of velocity field across the surface and rigid boundary), one can obtain ${p}_{n}^{\pm }$ and corresponding field distribution in the region *n*. The reflection coefficient is

2 $$r=\frac{{p}_{0}^{-}}{{p}_{0}^{+}}=\frac{{\rho }_{1}{k}_{0y}-i\,\tan ({k}_{1y}d){\rho }_{0}{k}_{1y}}{{\rho }_{1}{k}_{0y}+i\,\tan ({k}_{1y}d){\rho }_{0}{k}_{1y}}.$$

To simplify the equation, we can take approximation $\tan ({k}_{1y}d)\approx {k}_{1y}d$ when $|{k}_{1y}d|\ll 1$. For the normal incidence case, we obtain

3 $$r=\frac{{\rho }_{1}{k}_{0}-i{k}_{1}^{2}d{\rho }_{0}}{{\rho }_{1}{k}_{0}+i{k}_{1}^{2}d{\rho }_{0}}$$

where ${{k}_{1}}^{2}={{k}_{0}}^{2}{E}_{0}{\rho }_{1}/({E}_{1}{\rho }_{0})$, and Eq. (3) reduces to

4 $$r=\frac{{E}_{0}^{-1}-i{E}_{1}^{-1}{k}_{0}d}{{E}_{0}^{-1}+i{E}_{1}^{-1}{k}_{0}d}.$$

The absorptance *α* of the system can be expressed as $\alpha =1-|r{|}^{2}-|t{|}^{2}$ with *r* and *t* representing the reflection and transmission coefficient, respectively. Due to the impedance mismatch between the air and the rigid wall, the transmission coefficient is *t* = 0. Thus, the PA can be achieved, if the reflected waves are almost canceled ($r\approx 0$). It is found that if ${E}_{1}/{E}_{0}=\frac{2\pi d}{\lambda }i$ (*λ* is the wavelength in the air) is satisfied, the reflection vanishes and thus the resultant PA is achieved. When ${E}_{1}/{E}_{0}=\frac{2\pi d}{\lambda }i$, $|{k}_{1y}d|\ll 1$ can be transformed into $\,|\frac{{\rho }_{1}d}{{\rho }_{0}\lambda }|\ll \frac{1}{2\pi }$, which denotes that the thickness of the MA slab should be small enough to meet the approximation condition.

For illustration, we use Eq. (2) to investigate the following three cases. For simplicity, we set ${\rho }_{0}={\rho }_{1}=1.21\,{\rm{kg}}/{{\rm{m}}}^{3}$ and *E*_0_ = 1.42 × 10^5^ Pa. Figure 1(b) shows the map of the reflection coefficient *R* with the real and imaginary parts of effective bulk modulus normalized to the background air at the thickness $d=\lambda /30$. It is observed that the PA condition can be achieved with *E*_1_/*E*_0_ = 0.209*i*. Any deviation of real (*E*_1_/*E*_0_) from zero would cause lower absorption and thus it is critical to maintain a vanishing real part of *E*_1_/*E*_0_ in order to achieve PA. For clarifying the statement, we have derived the reflection spectrum of the MA for the different values of the thickness *d* with *E*_1_/*E*_0_ = 0.041*i*, 0.209*i* and 0.758*i*, as shown in Fig. 1(c). The quotient of *E*_1_/*E*_0_ and *d* at the absorptive peak conforms to $\frac{{{\rm E}}_{1}}{{{\rm E}}_{0}d}=\frac{2\pi }{\lambda }i$, which satisfies the PA conditions. In these three cases, the highest absorption is achieved for the thinnest case with the *λ* fixed. The map of the reflection coefficient *r* with the imaginary part of the normalized effective bulk modulus and the thickness is shown by Fig. 1(d). Moreover, for generality, the effective bulk modulus for achieving PA at different *d*/*λ* (with *λ* fixed) has been illustrated by the dashed line, which is consistent with the absorptive peaks.

### Numerical simulations

**Figure 2:**
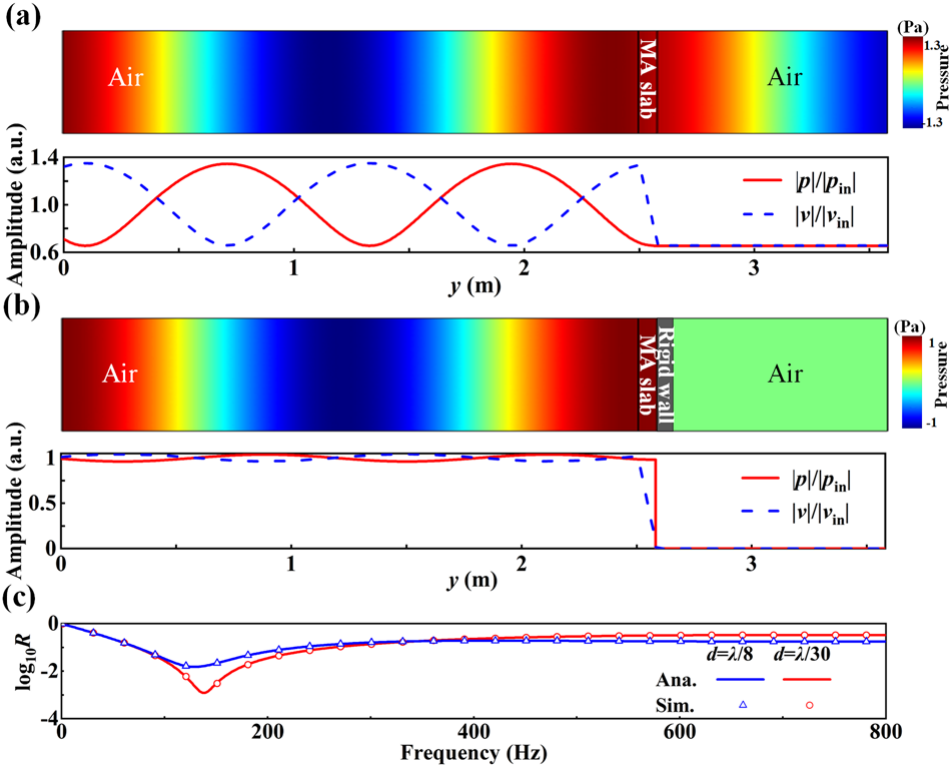
Normalized amplitudes of pressure (red solid lines) and velocity (blue dashed lines) for cases of (**a**) an ultra-thin MA film with imaginary bulk modulus in air, (**b**) a rigid wall on the right side of the film. (**c**) Analytical and simulated reflection coefficients for the two cases at different values with (*d*, *E*_1_/*E*_0_ = (*λ*/30,0209*i*)) and (*λ*/8, 0.785*i*).

To verify the analytical results, we perform numerical simulations, as shown in Fig. 2. The sound waves at 139 Hz (with a wavelength of 2.49 m) are incident from the left side onto an ultra-thin homogenous MA slab with a thickness of *d* = 0.08 m (~*λ*/30), a density of ${\rho }_{1}=1.21\,{\rm{kg}}/{{\rm{m}}}^{3}$, and a normalized effective bulk modulus *E*_1_/*E*_0_ = 0.209*i*. In Fig. 2(a,b), we show the pressure distribution, normalized amplitudes of pressure $|p|/|{p}_{{\rm{in}}}|$ (red solid lines) and particle velocity $|v|/|{v}_{{\rm{in}}}|$ (blue dotted lines) without and with a rigid backing boundary, respectively, where *p*_in_ and *v*_in_ correspond to the normal pressure and the normal particle velocity of incident waves. In the case without the rigid boundary, the reflection and transmission coefficients of Fig. 2(a) can be calculated by

5 $$r^{\prime} =\frac{{e}^{i{k}_{1}d}-{e}^{-i{k}_{1}d}}{(1+{\zeta }_{01})(1+{\zeta }_{10}){e}^{i{k}_{1}d}+(1-{\zeta }_{01})(1-{\zeta }_{10}){e}^{-i{k}_{1}d}},$$

6 $$t^{\prime} =\frac{4}{(1+{\zeta }_{01})(1+{\zeta }_{10}){e}^{i{k}_{1}d}+(1-{\zeta }_{01})(1-{\zeta }_{10}){e}^{-i{k}_{1}d}},$$

where ${\zeta }_{01}=\frac{{\zeta }_{1}}{{\zeta }_{0}}$, ${\zeta }_{10}=\frac{{\zeta }_{0}}{{\zeta }_{1}}$, and ${\zeta }_{0}$ (${\zeta }_{1}$) is the impedance of the air (metamaterial). In this case, less than half of the sound energy (with *α* = 45%) can be absorbed. It is observed that the pressure is almost a constant across the slab, while an abrupt change takes place in the particle velocity due to the purely imaginary bulk modulus. Note that in the impedance-match case^39^ without the rigid boundary, the relative density should be $\frac{{\rho }_{1}}{{\rho }_{0}}=\frac{{E}_{0}}{{E}_{1}}=-\,\eta i$ ($\eta$ is for the dissipation loss factor), i.e., a double-zero MA is harder to be implemented in experimental realization. In the impedance-match case, Eqs (5 and 6) can be simplified to *r* = 0 and $t={e}^{-i2\pi \eta d/\lambda }$, while the energy transmission coefficient *T* is given by $T=-\,55\eta d/\lambda$ (dB). To achieve the same absorption level (−30 dB) as our case of the MNZ MA, the slab thickness *d* should be *λ*/9 for the impedance-matched MAs with the same loss ($\eta =4{\rm{.8}}$). Thus, the MNZ MA can be 70% thinner than the MAs without the rigid boundary.

However, almost all the incident waves in Fig. 2(b) are absorbed with the ultra-thin slab backed by a rigid wall. There is almost no reflection, which can be deduced from the nonexistence of variance (see Fig. 2(b)) in the normalized amplitudes in the background region. A simplified analysis^40^ of the total reflection by this structure considers only the interferences between the direct reflection from the metamaterial, and the multiple reflections between the metamaterial and the rigid wall, which gives

7 $$r=r^{\prime} +\frac{t{\text{'}}^{2}{e}^{2i{k}_{1}d}}{1-r\text{'}{e}^{2i{k}_{1}d}}.$$

When the incident plane waves impinge on the surface of the ultra-thin slab, a direct reflection component *p*_0_ occurs, which brings a π shift. The waves through the slab are reflected by the rigid boundary, which have a zero phase change. A part of waves is absorbed while the other part of it, denoted by component *p*_1_, is multi-refracted out of the surface. Component *p*_0_ is π out of phase with component *p*_1_. Consequently, the rigid wall not only plays a role in suppressing the transmission but also reduces the reflection due to destructive interference. The resultant surface impedance matches with the air. The reflection spectrum in Fig. 2(c) is calculated for two cases with different values of slab thickness *d* and bulk modulus *E*_1/_*E*_0_. The quotient of *d* and *E*_1_ is fixed to ${E}_{1}/{E}_{0}=\frac{2\pi d}{\lambda }i$ to fulfill the condition of PA. The higher absorption (−30 dB, i.e., 99.9%) is obtained for the thinner case. Therefore, the above numerical simulations exactly agree with the analytical results.

### Design of the MNZ MA

**Figure 3:**
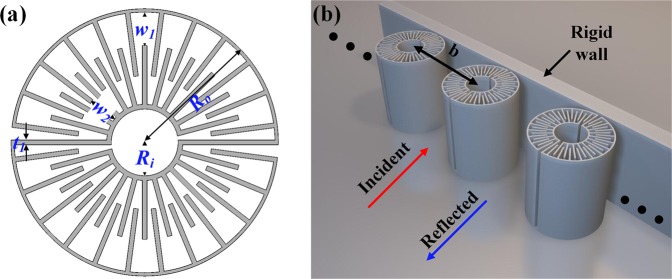
(**a**) Designed metamaterial units. (**b**) Schematics of the absorptive system consisting of a metamaterial layer backed by a rigid wall.

According to the above analysis, we have designed a practical metamaterial structure for satisfying the PA condition to construct a perfect absorber. The schematic cross-sectional view of the metamaterial structure, which exhibits zero bulk modulus, is shown in Fig. 3(a). The outer and inner radii of the MAs are *R*_*o*_ and *R*_*i*_, respectively. The structure is uniformly separated into two parts, with each part having a zigzag channel in an interdigital manner with the outer slit width ${w}_{1}$, the inner slit width ${w}_{2}$, and the wall thickness *t*. We continue to present an absorption system, comprising the metamaterial in a single configuration backed by a rigid wall (see Fig. 3(b)). As scalar waves, Sound waves propagate along the zigzag channels instead of in a straight line from the exterior to the central interconnection core, which enables the propagation length of the sound waves to be multiplied. Thus, the structure possesses a high relative refractive index *n*_r_ relative to the background medium. This unique characteristic can enable the artificial spatial concentration of sound in resonant patterns. The collective in-phase propagation inside the zigzag channels can enhance monopolar resonance. Our structure is robust in a wide range of periods (e.g. A > 0.9 for 89 mm~265 mm), which can be arranged sparsely in practical applications.

**Figure 4:**
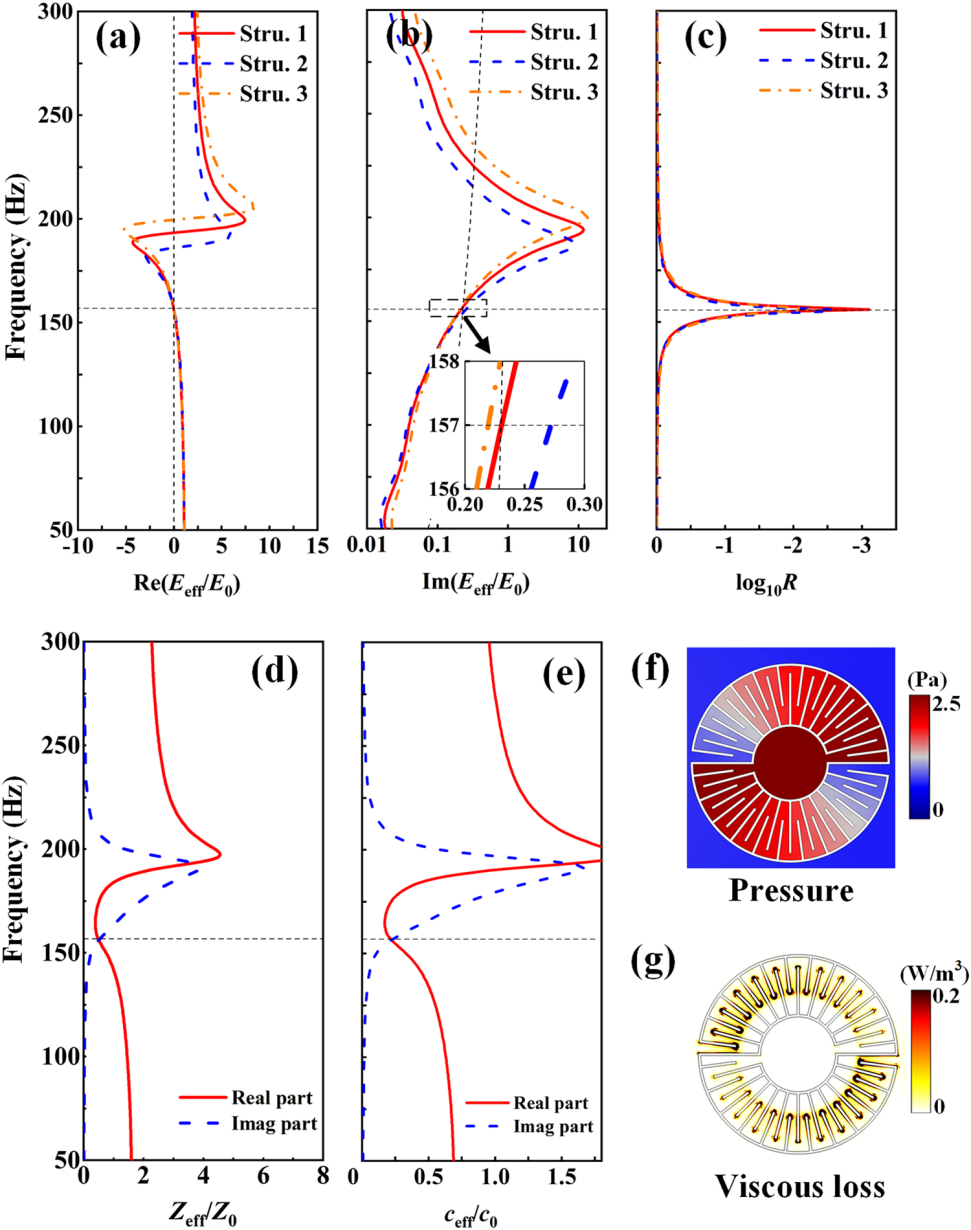
Retrieved parameters of Stru. 1, Stru. 2, and Stru. 3: (**a**) Real and (**b**) imaginary part of the effective bulk modulus. (**c**) Reflection coefficient of different structures. Retrieved parameters of Stru. 1: (**d**) effective impedance, and (**e**) effective sound velocity. (**f**) Sound pressure and (**g**) viscous loss of Stru. 1 at the absorptive peak.

The effective dynamic parameters are analyzed with thermo-viscous dissipation taken into consideration. As the working wavelength is much longer than the dimensions of the absorber and hence, it can be equivalent to be a homogenous plate according to the effective medium theory. The effective thickness here equals the diameter of the structure in Fig. 3(a). The effective dynamic parameters normalized to the background air can be retrieved by the transmission and reflection coefficients for a monolayer of the MAs. Here, we selected three structures to conduct the analysis. The retrieved bulk modulus *E*_eff_ is shown in Fig. 4(a,b) and the corresponding reflection spectra are shown in Fig. 4(c). The geometry parameters of structure 1, structure 2, and structure 3 are listed in Table 1. At 157 Hz, the real parts (see Fig. 4(a)) of the effective bulk modulus of structure 1, 2 and 3 all approach zero because the monopolar resonance occurs, while the imaginary parts (see Fig. 4(b)) of the effective bulk modulus differ from each other due to different widths of the channels. The imaginary parts of bulk modulus are 0.230*i*, 0.271*i*, and 0.219*i*, respectively. It is found that only structure 1 is set up with an appropriate value to fulfill the PA condition. By adjusting the parameters of the structures (w1, w2, Ri), i.e., the width of the zigzag channels, we can control the loss of the MA. From Fig. 4(c), it is found that the structure 1 has the highest absorption level (−31 dB in reflection) than the others, which is consistent with the analytical and simulated results. A remarkable absorptive peak with a value of 99.9% in the numerical-simulation prediction, i.e., nearly PA, accompanied by a significant cancel in the reflectance, can be seen at 157 Hz. This critical frequency corresponds exactly to the vanishing real part bulk modulus (see Fig. 4(a)). Shown by Fig. 4(a,b), the resonant response is highly excited as seen from the sharply-varied effective bulk modulus. Furthermore, we calculate the effective parameters of structure 1 shown by Fig. 4(d,e) to clarify the physical mechanism in the way of the equivalent medium. Figure 4(d) demonstrates an imaginary part of the effective impedance, which contributes to the high concentration of sound energy, while Fig. 4(e) shows an imaginary part of sound velocity which leads to the dissipation of sound waves. Hence, the MNZ MAs demonstrate intensive resonances to highly localize (due to the large imaginary part of effective impedance) and further dissipate (due to the large imaginary part of effective velocity) the sound energy inside the MA, which reveal the mechanism of absorptive peaks.

**Table 1:** Geometric parameters of the MNZ Mas.

	*R*_*o*_ (mm)	*t*_1_ (mm)	*w*_1_ (mm)	*w*_2_ (mm)	*R*_*i*_ (mm)	*b* (mm)	*E*_eff_/*E*_0_ (a.u.)
Stru. 1	40	1	3.5	9.1	17	133	0.230*i*
Stru. 2	40	1	3	10.8	15	133	0.271*i*
Stru. 3	40	1	4	10.1	15	133	0.219*i*

The detailed procedures to determine the parameters are as follows: We fix the working frequency, the thickness *d* = 2*R*, and period *b*. Next, by scanning the parameters (*w*_1_, *w*_2_, *R*_*i*_) and frequency, the terms *E*_eff_/*E*_0_ can be derived and we further determine the values of *w*_1_, *w*_2_ and *R*_*i*_, the MNZ frequency satisfying ${E}_{{\rm{eff}}}/{E}_{0}=\frac{2\pi d}{\lambda }i$. Finally, we select the results with the highest absorption.

To exploit the physical mechanism of the PA intuitively, the pressure distribution and thermo-viscous dissipation distribution at the absorptive peak are analyzed. The pressure distribution (see Fig. 4(f)) demonstrates the monopolar nature of resonant in the MA. The sound energy is concentrated in the maze-like structure. The thermo-viscous dissipation density distribution (see Fig. 4(g)) show that the sound energy is largely dissipated by friction loss in the narrow region of the MAs, which explains that the viscous loss can be adapted by changing the width of the zigzag channel.

### Experiment

**Figure 5:**
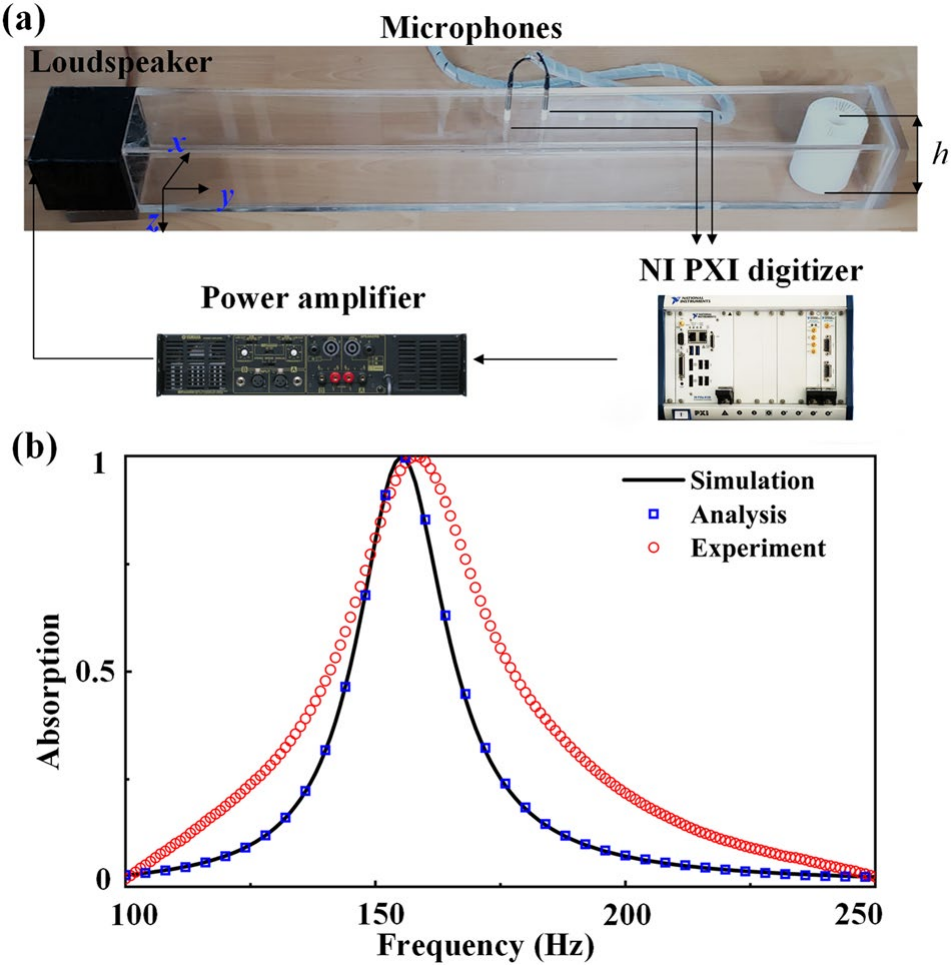
(**a**) Measurement system of sound absorptive coefficients. (**b**) Simulated and measured absorption coefficients (>99%) around the frequency of 157 Hz. The measured results agree with the simulated results.

Figure 5(a) shows the configuration of the measurement system constructed in a rectangular impedance tube with side lengths of *l*_*z*_ = 100 mm and *l*_*y*_ = 133 mm. The MA sample of height *h* = 100 *mm* is located at the terminal of the tube backed by a hard block. A loudspeaker is mounted on the input surface of the acoustic impedance tube to generate incident plane waves. The measured absorption is shown as red circles in Fig. 5(b), which are nearly consistent with the simulated results (blue solid line) and the analytical results (orange solid symbols) calculated by using the Eq. (2) with retrieved parameters. The limited rigidity of the sample fabricated with epoxy resin could account for the minor deviation (~2 Hz) of the absorptive peak. The consistency confirms the effectiveness of the equivalent model. Therefore, the experimental results confirm that the PA can be achieved with MNZ metamaterials.

In the proposed structure, the geometric parameters are configured to achieve the required purely imaginary bulk modulus (${E}_{{\rm{eff}}}/{E}_{0}=\frac{2\pi d}{\lambda }i$) according to our MNZ theory while the period is fixed. In contrast, the geometric parameters of the Mie resonator in ref.^38^ are fixed (with fixed loss factor) and the near critical coupling condition is satisfied by tuning the periods of Mie resonators (changing leakage factor). Our theory can also be applied in explaining the mechanism of the absorption of the double-channel Mie resonator (DMR). At low-order monopole mode, i.e., the fundamental monopole, the resonant response is highly excited as seen from the sharply-varied effective bulk modulus. Moreover, the effective bulk modulus demonstrates a large imaginary part. At the first absorptive peak, the real part of bulk modulus of DMR approaches zero, and the relative value of the imaginary part is 0.2575*i*. The analytical absorption coefficient calculated by using Eq. (2) with the above retrieved parameters is *α* = 0.98, which is consistent with the simulated result.

## Discussion

In summary, inspired by the conception of permeability-near-zero metamaterials in electromagnetic waves, a theory of the PA of MNZ metamaterials has been proposed in the acoustic regime. We analytically deduce and numerically demonstrate the absorption of sound waves in ultra-thin slabs with imaginary bulk modulus backed by a rigid boundary. Based on the analytical equation, the absorption is dependant on the thickness and bulk modulus of the MAs. We utilize an ultra-thin metamaterial, of which the feasible constitutive parameters can be derived from employing the appropriate configuration parameter. Moreover, the MNZ theory can serve as an explanation for the absorption of the other case in a way. The design idea of the MNZ MAs can be straightforwardly spread to the optimal design of sound absorption in the propagation path. Compared to previous metamaterials, our structure possesses an advantage in high sparsity, in addition to easy fabrication in 3D printing and good robustness in practical applications.

## Methods

### Simulation

Throughout the paper, the numerical simulations of Finite Element Analysis (FEA) are performed by the commercial finite element package COMSOL Multiphysics. The simulations in Fig. 2 are performed in the Pressure Acoustic module with the effective medium. Perfectly matched layers are imposed on the exterior of the air domain in the x direction to eliminate interference from the reflected waves. Periodic boundary conditions are set in the y direction. Simulations in Fig. 4 are calculated by using the Acoustic-Thermoacoustic Interaction and Frequency domain module in COMSOL Multiphysics. The dynamic viscosity of air (${\mu }_{{\rm{d}}}=1.81\times {10}^{-5}$ Pa s) is used to characterize the loss factor.

### Experiment

The impedance tube is made of plexiglass, and the MA sample is fabricated with epoxy resign (with a mass density of *ρ*_*e*_ = 1050 kg/m^3^ and a sound velocity of *c*_*e*_ = 2200 m/s) by 3D printing technology. We adopt the standard test method (ASTM E1050–12) to conduct the measurement. We use two 1/4 inch condensed microphones (Brüel&Kjær 4939) to record sound pressure and employ a digitizer (NI PXle-8135) to acquire sound pressure for processing in the LabVIEW program.

## Data Availability

The datasets generated during and/or analyzed during the current study are available from the corresponding author on reasonable request.
